# Supplementary material for: Modal-based estimation via heterogeneity-penalized weighting: model averaging for consistent and efficient estimation in Mendelian randomization when a plurality of candidate instruments are valid
Source: Int J Epidemiol. 2018 May 26;47(4):1242–54. doi: 10.1093/ije/dyy080 (PMC6124628; doi:10.1093/ije/dyy080)
Supplement: Supplementary Data [file dyy080_supplementary_material.pdf]

Supplementary Material

A.1 Software code

```
###### Summarized data set-up
bx   # genetic associations with risk factor
by   # genetic associations with outcome
bxse # standard errors of genetic associations with risk factor
byse # standard errors of genetic associations with outcome
######
bx  =c(0.160, 0.236, 0.149, 0.09, 0.079, 0.072, 0.047, 0.05, 0.069,
    0.039, 0.088, 0.032, 0.104, 0.045, 0.054, 0.032, 0.032)
by  =c(0.0237903, -0.1121942, -0.0711906, -0.030848, 0.0479207, 0.0238895,
  0.005528, -0.0327605, 0.0214852, -0.0387675, -0.0304042, -0.0082261,
  0.0246432, 0.0148795, -0.0498487, 0.0155667, 0.0242003)
bxse=c(0.006, 0.009, 0.006, 0.005, 0.005, 0.005, 0.006, 0.006, 0.011,
  0.006, 0.015, 0.006, 0.015, 0.007, 0.009, 0.006, 0.007)
byse=c(0.0149064, 0.0303084, 0.0150552, 0.0148339, 0.0143077, 0.0145478,
  0.0160765, 0.0140347, 0.0255237, 0.0139256, 0.0441698, 0.0162031,
  0.0444987, 0.016674, 0.0220043, 0.018098, 0.0219547)
  # example data (CRP-CAD associations)
  #
### Simple (but inefficient) code
  #
library(R.utils)
pen.weight <- function(theta, thetase, thetamean) {
 return( exp(-sum(log(thetase))-sum((theta-thetamean)^2/thetase^2/2)) ) }
  # this is the heterogeneity penalty weighting function
post=NULL; est=NULL; seest=NULL
  # these are the heterogeneity-penalized weights and means and standard deviations
  #   of the normal distributions in the weighted mixture distribution
for (i in 1:(2^length(bx)-1)) {
  inc=as.numeric(strsplit(intToBin(i),"")[[1]])
  inc=c(rep(0,length(bx)-length(inc)), inc)
  prior = ifelse(sum(inc)<1.5, 0, 1)
   # prior is set to zero for all models with 0 or 1 variants,
   #   equal for all other subsets
  estinc  = (by/bx)[which(inc==1)]
  seinc   = abs((byse/bx)[which(inc==1)])
  meaninc = sum(estinc*seinc^-2)/sum(seinc^-2)
  weight = pen.weight(estinc, seinc, meaninc)
  post[i] = prior*weight
  est[i]  = meaninc
  if (sum(inc) > 1) {
 seest[i] = summary(lm(by[which(inc==1)]~bx[which(inc==1)]-1,
             weights=byse[which(inc==1)]^-2))$coef[1,2]/
       min(summary(lm(by[which(inc==1)]~bx[which(inc==1)]-1,
             weights=byse[which(inc==1)]^-2))$sigma, 1)
             }
  if (sum(inc) == 1) {
```

```
 seest[i] = byse[which(inc==1)]/bx[which(inc==1)] }
 }
post.norm = post/sum(post)
 # normalized heterogeneity-penalized weights
sumlik=NULL
point = seq(from=-1, to=1, by=0.001)
for (i in 1:length(point)) {
 lik = post.norm*dnorm(point[i], mean=est, sd=seest)
 sumlik[i] = sum(lik) }
  # calculates the likelihood at a range of values from -1 to +1
  # if the causal effect may be outside of this range,
  #  then this range of values will need to be expanded
whichin = which(2*log(sumlik)>(2*max(log(sumlik))-qchisq(0.95, df=1)))
  # provides an index of estimate values in the 95% confidence interval
estimate = -1.001+0.001*which.max(log(sumlik))
  # modal estimate
ifelse(sum(diff(whichin)!=1)==0, "Single range", "Multiple ranges")
  # returns "Single range" if the 95% CI is a single range of values
  # returns "Multiple ranges" otherwise
lowerCI    = -1.001+0.001*whichin[1]
upperCI    = -1.001+0.001*whichin[length(whichin)]
  # lower and upper confidence interval limits (assuming single range)
fullCI     = -1.001+0.001*whichin
  # all estimate values in confidence interval
  # if the likelihood is calculated for a different range of values (not -1 to +1),
  #  then this code will need to be altered
  #
  #
### Efficient (but harder to follow) code
  #
library(iterpc);
library(Matrix);
  #
model.prior = function(model.size, N.obs, prob.valid.inst){
  pr = (prob.valid.inst^model.size)*(1-prob.valid.inst)^(N.obs-model.size)
  return(pr)
}
  #
het.weight = function(prob.valid.inst, bx, by, byse){
  J = length(by);
  theta.est = by/bx;
  theta.se = abs(byse/bx);
  tmp.1 = by/byse;
  tmp.2 = bx/byse;
  theta.se.sq = theta.se^2;
  log.theta.se = log(theta.se);
  est  = seest = vector("numeric", 2^J-1);
  het.weight = vector("numeric", 2^J-1);
   #
```

```
  count = 0;
 for(n in 1:J){
    perms = choose(J,n);
    inc = sparseMatrix(i=as.vector(t(replicate(n,1:perms))),
                       j=as.vector(t(getall(iterpc(J,n,c(1:J))))),
                       x=1, dims = c(perms,J));
    # sparse binary inclusion matrix
    # 1 denotes an instrument is included in the model
    # each row represents a particular model
    est.sum = inc%*%(theta.est/theta.se.sq);
    recip.var.ivw = inc%*%(1/theta.se.sq);
    est.ivw = est.sum/recip.var.ivw;
    est[(count+1):(count+perms)] = est.ivw;
  if(n>1){
     tmp = t(replicate(J, as.vector(est.ivw)));
   if(n<J){
     psi.hat = sqrt((1/(n-1))*rowSums(t(t(inc)*(tmp.1^2 - 2*tmp*(tmp.1*tmp.2) +
                    (tmp^2)*(tmp.2^2)))))
           }
      else{
        psi.hat = sqrt((1/(n-1))*sum(tmp.1^2 - 2*tmp*(tmp.1*tmp.2) +
                    (tmp^2)*(tmp.2^2)));
           }
      psi.hat[which(psi.hat<1)] = 1;
      seest[(count+1):(count+perms)] = psi.hat/sqrt(recip.var.ivw);
          }
  else if(n==1){
      seest[(count+1):(count+perms)] = inc%*%theta.se;
                }
     #
  if(n>1){
      het.exponent = rowSums(inc*t(t(t(inc)*theta.est) -
            as.vector(est.ivw))^2/theta.se.sq));
      het.weight[(count+1):(count+perms)] =
                 exp(-(inc%*%(log.theta.se)+0.5*het.exponent))*
                 model.prior(n,J,prob.valid.inst);
          }
    count = count+perms;
  }  # ends for loop
  newlist = list(het.weight, est, seest);
  return(newlist)
}
   #
results = het.weight(0.5, bx, by, byse);
het.weight = results[[1]];
het.weight.norm = het.weight/sum(het.weight);
# normalized heterogeneity-penalized weights
est = results[[2]];
seest = results[[3]];
```

```
   #
sumlik=NULL
grid.increment = 1e-3; grid.start = -1; grid.end = 1;
point = matrix(seq(grid.start, grid.end, grid.increment), ncol = 1);
   #
l = length(het.weight.norm);
sumlik = vapply(point,function(i){sum(het.weight.norm*dnorm(rep(i,l), est, seest))}, 1);
   # calculates the likelihood at a range of values from -1 to +1
   # if the causal effect may be outside of this range,
   #  then this range of values will need to be expanded
whichin = which(2*log(sumlik)>(2*max(log(sumlik))-qchisq(0.95, df=1)));
   # provides an index of estimate values in the 95% confidence interval
estimate = -1.001+0.001*which.max(log(sumlik));
   # modal estimate
ifelse(sum(diff(whichin)!=1)==0, "Single range", "Multiple ranges");
   # returns "Single range" if the 95% CI is a single range of values
   # returns "Multiple ranges" otherwise
lowerCI    = -1.001+0.001*whichin[1];
upperCI    = -1.001+0.001*whichin[length(whichin)];
   # lower and upper confidence interval limits (assuming single range)
fullCI     = -1.001+0.001*whichin;
```

A.2 Technical motivation of model averaging method

We construct a likelihood function for the causal effect θ. We assume that at least two of the genetic variants are valid instruments. Therefore, there exists one subset of genetic variants containing all the valid instruments and no invalid instruments. Following the principles of likelihood-based inference (the conditionality and sufficiency principles), we base our inferences on all variants in this subset, and in particular on the IVW estimate corresponding to this subset (which is a sufficient statistic for that model). We define a classification variable C, which takes values that correspond to the subsets of candidate instruments; $C = k$ is the event that subset k contains all the valid instruments and no invalid instruments. We take the IVW estimates based on the subsets of variants and their standard errors as the data in our estimation procedure. We further assume that the standard errors are known without error. For convenience, we denote the vector of IVW estimates as $\mathbf{A_1}$, and the vector of standard errors as $\mathbf{A_2}$. Conditional on the kth subset containing valid instruments, the IVW estimate is normally distributed with mean θ and variance equal to the square of its standard error. This enables us to consider a likelihood function for θ as:

$$L(\theta) = f(\mathbf{A_1}, \mathbf{A_2}; \theta) \tag{A1}$$

where $f(\mathbf{A_1}, \mathbf{A_2}; \theta)$ is the probability density function for the IVW estimates. We then condition on the identity of the subset containing all the valid instruments C:

$$\begin{aligned}
L(\theta) &= f(\mathbf{A_1}, \mathbf{A_2}; \theta) && \text{(A2)}\\
&= \sum_k f(\mathbf{A_1}, \mathbf{A_2}; \theta | C = k)\mathbb{P}(C = k) && \text{(A3)}\\
&\propto \sum_k w'_k a_{2k}{}^{-1} \exp\left[-\frac{(\theta - a_{1k})^2}{2a_{2k}{}^2}\right] && \text{(A4)}\\
&\propto \sum_k w'_k \operatorname{se}(\hat{\theta}_{IVWr,k})^{-1} \exp\left[-\frac{(\theta - \hat{\theta}_{IVW,k})^2}{2\operatorname{se}(\hat{\theta}_{IVWr,k})^2}\right] && \text{(A5)}
\end{aligned}$$

where $w'_k = \mathbb{P}(C = k)$ is the weight of model k. This likelihood function is the function on which we base our inferences for the heterogeneity-penalized model averaging method.

The weights are derived using a distance measure that promotes the inclusion of multiple genetic variants, but penalizes heterogeneity. Our formula for deriving weights can be viewed as a Bayesian inference method in which the prior probabilities of the subsets are all equal. The posterior weights w'_k are calculated as the prior multiplied by the likelihood assuming the variant-specific ratio estimates $\hat{\theta}_j$ are normally distributed about a common mean $\hat{\theta}_{IVW,k}$ with variant-specific standard deviation $\operatorname{se}(\hat{\theta}_j)$ for all variants j in subset k:

$$w'_k \propto \prod_{j\in\sigma_k} \operatorname{se}(\hat{\theta}_j)^{-1} \exp\left[-\frac{(\hat{\theta}_j - \hat{\theta}_{IVW,k})^2}{2\operatorname{se}(\hat{\theta}_j)^2}\right]. \tag{A6}$$

where σ_k is the kth subset and $j \in \sigma_k$ loops across all genetic variants in the kth subset. As they based on a distance metric, these weights are independent of the value of θ, and so C is an ancillary variable (justifying the use of C in the conditionality principle).

A.3 Additional simulation: 6 invalid instruments

We conducted an additional simulation with 6 invalid instruments, and two different sample sizes. First, we estimated genetic associations based on 20 000 individuals for the gene–risk factor and gene–outcome associations. Secondly, we estimated genetic associations based on 100 000 individuals; a five-fold increase in sample size. Results are shown in Supplementary Table A1. We see that mean estimates for the inverse-variance weighted and weighted median estimates change only slightly with the increased sample size. There is a slight reduction in weak instrument bias with a positive causal effect, but otherwise bias is unaffected for these methods and Type 1 error rates are worse with the increased sample size. In contrast, bias for the heterogeneity-penalized method reduces by around a half as the sample size increases, and Type 1 error rates drop markedly. This suggests that while the asymptotic result is that bias should reduce to zero when a plurality of genetic variants are valid instruments, with finite sample sizes it is still important to pay attention to instrument validity. By comparison, while estimates from the MBE method (simple and weighted, $\phi = 1$, not assuming NOME) are similar to those from the heterogeneity-penalized method with the original sample size (none of the three methods dominates the other two across the scenarios either in terms of bias, Type 1 error rate or power), estimates from the MBE method improve somewhat with respect to bias, but not particularly with respect to Type 1 error rate. This suggests that the convergence properties are better for the heterogeneity-penalized method than for the MBE method.

	Original sample size			Increased sample size		
Method	Scenario 2	Scenario 3	Scenario 4	Scenario 2	Scenario 3	Scenario 4
Null causal effect: $\theta = 0$						
Inverse-variance weighted	0.001 (8.2)	0.410 (68.4)	0.271 (44.5)	−0.002 (8.6)	0.424 (83.5)	0.278 (49.1)
Weighted median	0.001 (20.9)	0.311 (59.1)	0.296 (71.4)	−0.001 (26.7)	0.296 (78.9)	0.293 (81.2)
Simple MBE	0.006 (8.9)	0.264 (22.3)	0.146 (21.8)	0.008 (14.8)	0.175 (32.7)	0.082 (20.1)
Weighted MBE	0.011 (11.8)	0.165 (21.7)	0.284 (61.0)	−0.001 (22.3)	0.121 (39.0)	0.270 (65.7)
Heterogeneity-penalized model averaging	0.003 (11.0)	0.228 (23.5)	0.210 (36.4)	0.000 (7.6)	0.088 (12.9)	0.097 (18.0)
Positive causal effect: $\theta = +0.2$						
Inverse-variance weighted	0.193 (17.5)	0.602 (95.8)	0.465 (78.4)	0.196 (18.7)	0.623 (99.9)	0.476 (83.8)
Weighted median	0.189 (38.2)	0.504 (89.9)	0.492 (99.0)	0.198 (77.0)	0.496 (99.8)	0.494 (99.0)
Simple MBE	0.207 (20.6)	0.473 (56.3)	0.354 (49.3)	0.212 (62.6)	0.384 (91.3)	0.290 (76.8)
Weighted MBE	0.154 (27.4)	0.339 (60.6)	0.455 (78.1)	0.172 (70.5)	0.310 (97.2)	0.451 (87.4)
Heterogeneity-penalized model averaging	0.192 (26.2)	0.423 (63.8)	0.417 (63.9)	0.197 (73.1)	0.288 (91.5)	0.312 (86.8)

Supplementary Table A1: Mean estimates (Type 1 error rate/power for 95% confidence interval [%]) for selected methods with 6 invalid instruments for original and increased sample sizes.

A.4 Additional simulation: clustered pleiotropy

We conducted another additional simulation in which some of the genetic variants had one magnitude of causal effect and others had a different magnitude. In particular, the causal estimate for 6 of the variants was zero (the majority causal effect), and the causal estimate for the other 4 variants was +1. The purpose of the simulation is to evaluate the performance of the model averaging method in a scenario that is likely to lead to uncertainty in which group of genetic variants is the modal group, and to a confidence interval that consists of two disjoint ranges for a considerable proportion of simulated datasets. Data were generated by setting the causal effect of the risk factor $\theta = 0$, but the pleiotropic effects $\alpha_j = +1 \times \gamma_j$ for the genetic variants with causal effects of +1. As the majority of genetic variants had a causal estimate of zero, we would expect the mean estimate from the model averaging method to be close to zero. We also conducted a simulation with 6 variants having a causal estimate of +1 and 4 having a causal estimate of 0 (majority causal effect $\theta = +1$).

Supplementary Table A2 shows mean estimates, median estimates, and empirical power of the 95% confidence interval (equivalent to the Type 1 error rate when the true majority causal effect is 0) for a selection of methods. Median estimates from the model averaging method are close to unbiased with a null causal effect. There is some attenuation in median and mean estimates with a positive causal effect. Mean estimates are somewhat different to median estimates, suggesting that the minority causal effect is estimated in a small number of simulated datasets – this is expected as in some cases, association estimates from the variants having the minority causal effect will be by chance more homogeneous than those having the majority causal effect, as hence receive a greater weight. The Type 1 error rate was close to the nominal 5% rate. This is despite 17.6% of the simulations under a null majority causal effect (28.5% for a positive causal effect) resulting in a 95% confidence interval that consists of at least two disjoint ranges. This simulation provides evidence that close to nominal coverage properties can be preserved even in the case that there is systemic uncertainty over the identity of the modal estimate (and a bimodal log-likelihood).

In comparison, estimates from the mode-based estimation (MBE) method was similar (although surprisingly low for the weighted MBE method with a positive majority causal effect). Estimates from other methods, including the weighted median method, were disappointing with considerable bias and highly inflated Type 1 error rates.

	Majority causal effect: $\theta = 0$			Majority causal effect: $\theta = +1$		
	Mean	Median	Type 1 error	Mean	Median	Power
Inverse-variance weighted	0.383	0.381	62.6	0.576	0.574	95.3
Weighted median	0.256	0.201	38.8	0.633	0.675	89.3
Simple MBE	0.127	0.066	7.8	0.746	0.817	71.8
Weighted MBE	0.067	0.037	6.8	0.299	0.173	35.1
Heterogeneity-penalized model averaging	0.115	0.034	7.1	0.724	0.853	58.7

Supplementary Table A2: Mean estimates (Type 1 error rate/power for 95% confidence interval [%]) for selected methods with two sets of variants having different magnitudes of causal effect on the outcome.

A.5 Additional details about applied examples

LDL-cholesterol and CAD example: To assess the causal effect of LDL-cholesterol on CHD risk, we used 8 genetic variants in separate gene regions each of which has been specifically linked with LDL-cholesterol (each either encodes a biologically relevant compound to LDL-cholesterol, or is a proxy for an existing or proposed LDL-cholesterol lowering drug). These gene regions are: *HMGCR* (proxy for statin treatment), *PCSK9* (proxy for PCSK9 inhibition), *NPC1L1* (proxy for ezetimibe), *APOB* (encodes biologically relevant apolipoprotein B), *ABCG5/G8* (bile acid sequestrant), *SORT1* (antisense oligonucleotide RNA inhibitor targeting this pathway currently under development), *APOE* (encodes biologically relevant apolipoprotein E), and *LDLR* (encodes biologically relevant LDL receptor). The specific choice of variant in each gene region to include in the analysis was based on the lead variant from the 2010 analysis of the Global Lipids Genetic Consortium [Teslovich et al., Biological, clinical and population relevance of 95 loci for blood lipids. Nature 2010; 466:707–713].

Supplementary Table A3 provides information about these variants, including the beta-coefficients and standard errors for their associations per additional copy of the effect allele with LDL-cholesterol (mmol/L) and CAD risk (log odds ratios), together with the causal estimates based on each of these variants (log odds ratios for CAD per 1 mmol/L increase in LDL-cholesterol).

rsid	Nearest gene	Effect allele	Association with LDL-c Beta (SE)	Association with CAD risk Beta (SE)	Causal estimate Estimate (SE)
rs12916	*HMGCR*	C	0.063 (0.005)	0.036 (0.009)	0.566 (0.150)
rs2479409	*PCSK9*	G	0.052 (0.006)	0.029 (0.010)	0.556 (0.200)
rs2072183	*NPC1L1*	C	0.030 (0.005)	0.014 (0.012)	0.451 (0.394)
rs1367117	*APOB*	A	0.105 (0.005)	0.041 (0.011)	0.393 (0.101)
rs4299376	*ABCG5/G8*	G	0.071 (0.005)	0.051 (0.010)	0.714 (0.147)
rs629301	*SORT1*	T	0.146 (0.005)	0.101 (0.011)	0.694 (0.078)
rs4420638	*APOE*	G	0.185 (0.007)	0.092 (0.014)	0.498 (0.076)
rs6511720	*LDLR*	G	0.181 (0.008)	0.125 (0.017)	0.693 (0.094)

Supplementary Table A3: Details of genetic variants, beta-coefficients (standard errors, SE) for associations with low-density lipoprotein cholesterol (LDL-c, mmol/L) and with coronary artery disease (CAD) risk (log odds ratios) taken from CARDIoGRAM consortium, and causal effect estimates (log odds ratio per 1 mmol/L increase in LDL-cholesterol) for 8 genetic variants.

Weights for the 8 LDL-cholesterol variants as shown in Supplementary Figure A1 (top panel). These weights are the reciprocals of the standard errors of the ratio estimates ($w_j = \frac{\hat{\beta}_{Xj}}{\text{se}(\hat{\beta}_{Yj})}$ for genetic variant j), and relate to the proportion of variance in the risk factor explained by each variant. We also display relative weights w'_k (normalized to sum to 1) for the $2^8 - 8 - 1 = 247$ subsets of genetic variants considered in the model averaging method (2^8 subsets in total minus 8 singletons minus the empty set) in Supplementary Figure A1 (bottom panel). While the intuition of the heterogeneity-penalized model averaging method is that consistent estimation requires a weighted plurality of the genetic variants to be valid instruments (a weighted version of Hartwig et al's ZEMPA assumption), the subset weights depend not only on the weights for the individual variants, but also on the variant-specific ratio estimates and their standard errors, meaning that weights are dynamic, rather than fixed (they also vary with the sample size). Hence, although in this case the estimate based on all 8 variants receives the greatest weight, it only does so because the ratio estimates from all variants are similar to each other. In practice, one never knows which genetic variants are valid instruments and which are not (otherwise, analysis would be restricted to the valid instruments), hence the precise condition for consistency is somewhat moot as it can never be verified. A genetic variant should only be included in a Mendelian randomization investigation if investigators truly believe that it has a chance of being a valid instrument.

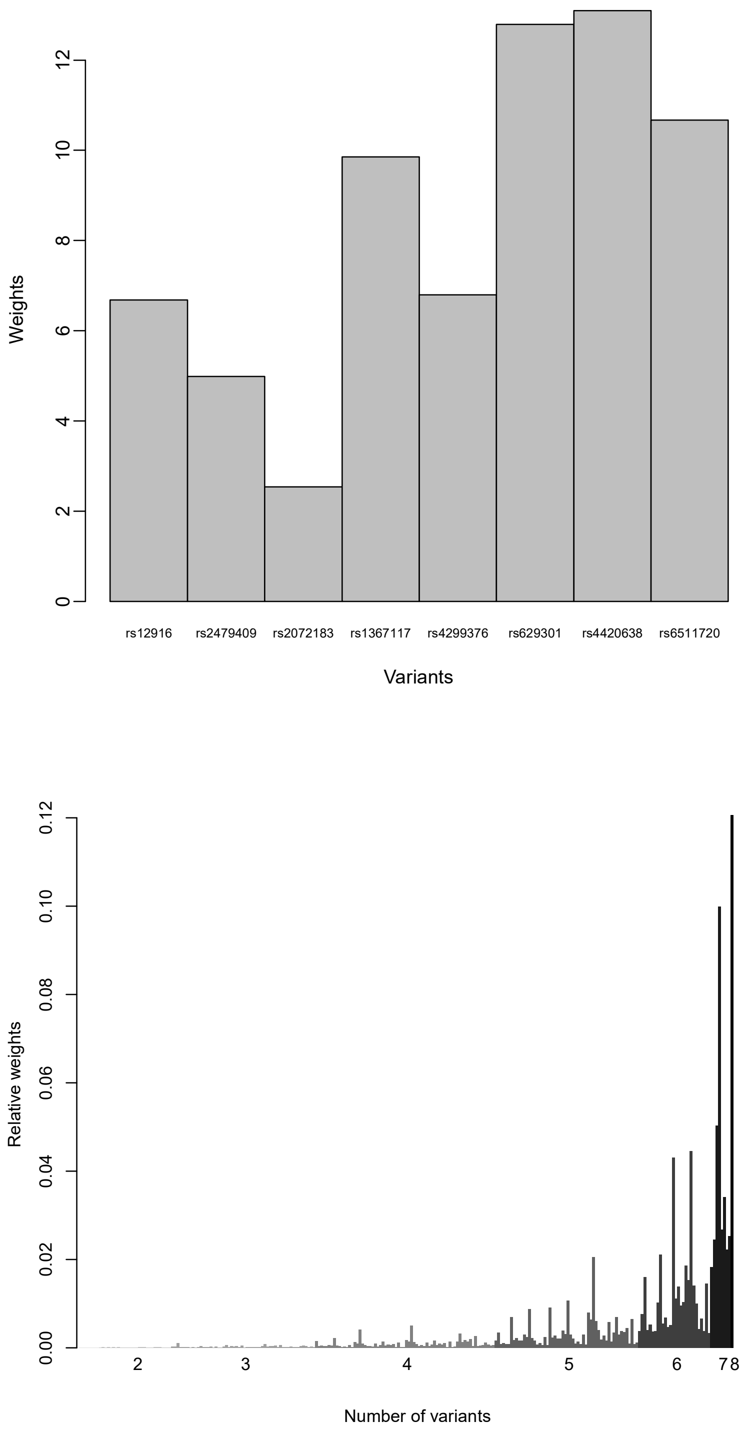

Supplementary Figure A1: Top panel: Weights for each of the 8 genetic variants. Bottom panel: Relative weights (normalized to sum to 1) for each of the subsets of variants. Subsets are arranged and coloured by the number of variants in that subset.

CRP and CAD example: Supplementary Table A4 provides information about the 17 variants used in the example analysis of this paper for investigating causal relationships between inflammation and coronary artery disease (CAD) risk, using C-reactive protein (CRP) as a measure of inflammation. All variants were previously demonstrated to be associated with CRP levels at a genome-wide level of significance by Dehghan et al. [Meta-analysis of genome-wide association studies in >80 000 subjects identifies multiple loci for C-reactive protein levels. Circulation 2011; 123(7):731–738]. Details of these variants are given, including the beta-coefficients and standard errors for their associations with CRP (log-transformed) and CAD risk (log odds ratios), together with the causal estimates based on each of these variants (log odds ratios for CAD per unit increase in log-transformed CRP).

rsid	Nearest gene	Effect allele	Association with CRP Beta (SE)	Association with CAD risk Beta (SE)	Causal estimate Estimate (SE)
rs2794520	*CRP*	C	0.160 (0.006)	0.024 (0.015)	0.149 (0.093)
rs4420638	*APOC1*	A	0.236 (0.009)	-0.112 (0.030)	-0.475 (0.128)
rs1183910	*HNF1A*	G	0.149 (0.006)	-0.071 (0.015)	-0.478 (0.101)
rs4420065	*LEPR*	C	0.090 (0.005)	-0.031 (0.015)	-0.343 (0.165)
rs4129267	*IL6R*	C	0.079 (0.005)	0.048 (0.014)	0.607 (0.181)
rs1260326	*GCKR*	T	0.072 (0.005)	0.024 (0.015)	0.332 (0.202)
rs12239046	*NLRP3*	C	0.047 (0.006)	0.006 (0.016)	0.118 (0.342)
rs6734238	*IL1F10*	G	0.050 (0.006)	-0.033 (0.014)	-0.655 (0.281)
rs9987289	*PPP1R3B*	A	0.069 (0.011)	0.021 (0.026)	0.311 (0.370)
rs10745954	*ASCL1*	A	0.039 (0.006)	-0.039 (0.014)	-0.994 (0.357)
rs1800961	*HNF4A*	C	0.088 (0.015)	-0.030 (0.044)	-0.346 (0.502)
rs340029	*RORA*	T	0.032 (0.006)	-0.008 (0.016)	-0.257 (0.506)
rs10521222	*SALL1*	C	0.104 (0.015)	0.025 (0.044)	0.237 (0.428)
rs12037222	*PABPC4*	A	0.045 (0.007)	0.015 (0.017)	0.331 (0.371)
rs13233571	*BCL7B*	C	0.054 (0.009)	-0.050 (0.022)	-0.923 (0.407)
rs2836878	*PSMG1*	G	0.032 (0.006)	0.016 (0.018)	0.486 (0.566)
rs4903031	*RGS6*	G	0.032 (0.007)	0.024 (0.022)	0.756 (0.686)

Supplementary Table A4: Details of genetic variants, beta-coefficients (standard errors, SE) for associations with C-reactive protein (CRP, log-transformed) and with coronary artery disease (CAD) risk, and causal effect estimates (log odds ratios for CAD per unit increase in log-transformed CRP) for 17 genome-wide significant variants.
